# Detection of CTX-M-15 beta-lactamases in Enterobacteriaceae causing hospital- and community-acquired urinary tract infections as early as 2004, in Dar es Salaam, Tanzania

**DOI:** 10.1186/s12879-017-2395-8

**Published:** 2017-04-17

**Authors:** Joel Manyahi, Sabrina J. Moyo, Marit Gjerde Tellevik, Faustine Ndugulile, Willy Urassa, Bjørn Blomberg, Nina Langeland

**Affiliations:** 1 Department of Clinical Science, University of Bergen, Bergen, Norway; 2 Department of Microbiology and Immunology, Muhimbili University of Health and Allied Sciences, Dar es Salaam, Tanzania; 3 National Centre for Tropical Infectious Diseases, Department of Medicine, Haukeland University Hospital, Bergen, Norway

**Keywords:** ESBL, Urinary tract infections, Tanzania

## Abstract

**Background:**

The spread of Extended Spectrum β-lactamases (ESBLs) among *Enterobacteriaceae* and other Gram-Negative pathogens in the community and hospitals represents a major challenge to combat infections. We conducted a study to assess the prevalence and genetic makeup of ESBL-type resistance in bacterial isolates causing community- and hospital-acquired urinary tract infections.

**Methods:**

A total of 172 isolates of *Enterobacteriaceae* were collected in Dar es Salaam, Tanzania, from patients who met criteria of community and hospital-acquired urinary tract infections. We used E-test ESBL strips to test for ESBL-phenotype and PCR and sequencing for detection of ESBL genes.

**Results:**

Overall 23.8% (41/172) of all isolates were ESBL-producers. ESBL-producers were more frequently isolated from hospital-acquired infections (32%, 27/84 than from community-acquired infections (16%, 14/88, *p <* 0.05). ESBL-producers showed high rate of resistance to ciprofloxacin (85.5%), doxycycline (90.2%), gentamicin (80.5%), nalidixic acid (84.5%), and trimethoprim-sulfamethoxazole (85.4%). Furthermore, 95% of ESBL-producers were multi-drug resistant compared to 69% of non-ESBL-producers (*p <* 0.05). The distribution of ESBL genes were as follows: 29/32 (90.6%) *bla*_CTX-M-15_, two *bla*_SHV-12_, and one had both *bla*_CTX-M-15_ and *bla*_SHV-12_. Of 29 isolates carrying *bla*_CTX-M-15_, 69% (20/29) and 31% (9/29) were hospital and community, respectively. *Bla*_SHV-12_ genotypes were only detected in hospital-acquired infections.

**Conclusion:**

*bla*_CTX-M-15_ is a predominant gene conferring ESBL-production in *Enterobacteriaceae* causing both hospital- and community-acquired infections in Tanzania.

**Electronic supplementary material:**

The online version of this article (doi:10.1186/s12879-017-2395-8) contains supplementary material, which is available to authorized users.

## Background

Extended Spectrum β-lactamases (ESBLs) have been observed in virtually all species of the family *Enterobacteriaceae*. Spread of ESBL-producing strains from general wards to intensive care units (ICU) and into the community can contribute to the further propagation of these resistant strains [1].

ESBLs are responsible for resistance against beta-lactam antibiotics such as penicillins, cephalosporins, monobactams and sometimes also carbapenems [2]. Organisms carrying ESBL enzymes often display co-resistance to other antibiotics including aminoglycosides, quinolones, trimethoprim-sulfamethoxazole and tetracycline [3, 4]. Spread of ESBL-producing bacterial isolates in the community has made empirical treatment of infections more difficult, and narrows the treatment options to expensive antibiotics like colistin and carbapenems.

Recent studies in Africa and Europe have found substantial increase in ESBL-producing Gram-negative bacteria causing community urinary tract infections, particularly harboring the *bla*_CTX-M-15_ allele [5–7]. Previously, studies in Tanzania have detected a substantial amount of ESBL-producing bacteria among the inpatients in intensive care and pediatric units in Tanzania [8, 9]. However, little is known regarding the frequency of ESBL-producers and ESBL genes in community-acquired (CA) and hospital-acquired (HA) urinary tract infections in Tanzania. A more recent study found a predominance of the *bla*_CTX-M-15_ genotype from human feces in a community setting [10].

Previous studies performed at Muhimbili National Hospital documented the presence of ESBL-producers, predominantly of the *bla*_CTX-M-15_ genotype, in hospital-acquired infections [8, 9]. Therefore, we decided to examine bacterial isolates collected prospectively for a period of six months in 2004 at Muhimbili National Hospital in Dar es Salaam, aiming at investigating whether ESBL-producers were present in the community setting, when hospital-acquired *bla*_CTX-M-15_ -producers were first reported in Tanzania. We also aimed to compare the ESBL genotypes circulating in the community to those found in the hospital setting.

## Methods

### Study setting and patient population

The study was conducted at Muhimbili National Hospital (MNH, Dar es Salaam, Tanzania. MNH is a tertiary health care facility that serves a population of about 4 million residents. The Department of Microbiology, MNH, receives samples from inpatients in the hospital wards, from the outpatient clinics and private health facilities in the city. Included in the study were samples from inpatients and outpatients with urinary tract infections seen at MNH between June 2004 and January 2005. Hospital-acquired infections were defined as those occurring in inpatients admitted at MNH for at least 72 h. Community-acquired infections were defined as those occurring in patients attending outpatient clinics at MNH. A UTI was defined as a positive urine culture of ≥10^5^ CFU/ml of pure bacterial growth.

### Clinical isolates

A total of 172 isolates of *Enterobacteriaceae* were collected, 84 and 88 isolates from hospital and community patients, respectively. All isolates were identified to the species level using established conventional procedures, the API 20E system (bioMérieux SA, Marcy l‵Etoile, France) or the Vitek 2 system (BioMérieux, Inc., Durham, N.C).

### Antimicrobial susceptibility testing

All isolates were tested for antimicrobial susceptibility using the disk diffusion method according to the Clinical & Laboratory Standards Institute’s guidelines [11]. The antimicrobials tested included amoxicillin/clavulanic acid (20/10μg), gentamicin (10μg), chloramphenicol (30μg), trimethoprim/sulfamethoxazole (1.25/23.75μg), doxycycline (30μg), nitrofurantoin(30μg), nalidixic acid (30μg), imipenem (10μg), ciprofloxacin (5μg), cefotaxime (30μg), ceftriaxone(30μg) and ceftazidime (30μg). Multidrug-resistant (MDR) bacteria were those bacteria which showed resistance to three or more classes of antimicrobial agents [12], classes including β-lactam/β-lactamase inhibitors, cephalosporins (ceftriaxone, ceftazidime, cefotaxime), aminoglycosides, fluoroquinolones (ciprofloxacin), tetracycline, cabepenems, Nitrofurantoin and trimethoprim-sulfamethoxazole. For ESBL isolates classes defining MDR excluded Penicillins and cephalosporins.

### ESBL detection

All isolates with reduced susceptibilities to ceftazidime (zone of inhibition < 22mm), ceftriaxone (zone inhibition <25 mm) and cefotaxime (zone inhibition <27 mm) disks according to CLSI guidelines [11], were tested for an ESBL using E-test ESBL strips as previously described [8] and PCR. Isolates with reduced susceptibilities to cephalosporin were confirmed ESBL by either using E-tests ESBL strips or PCR.

### Detection and identification of ESBL genotypes

All strain with reduced susceptibility to cephalosporin were examined for the presence of the *bla*_TEM_, *bla*_SHV_ and *bla*_CTX-M_ genes by PCR, using genomic DNA isolated by boiling. For *bla*_TEM_ amplification the primers described by Dubois et al. were used [13]. The cycling conditions were 95°C for 15 min, followed by 30 cycles of denaturation at 95°C for 1 min, annealing at 55°C for 1 min, elongation at 72°C for 1 min, followed by a final extension of 72°C for 10 min.

For *bla*_SHV_ amplification the primers SHV-1F (5′ – CGG CCT TCA CTC AAG GAT G – 3′) and SHV-1R (5′ – CGG STT AGC GTT GCC AGT – 3′) were used. The cycling conditions were the same as for those of blaTEM, but with an annealing temperature of 60°C. In PCR amplification targeting the *bla*CTX-M gene the primer pairs described by Pagani et al. [14] were used. The cycling conditions for these two primer pairs were initial activation at 95°C for 15 min, followed by 30 cycles of denaturation at 95°C for 30 s, annealing at 50°C for 40 s, elongation at 72°C for 1 min, followed by a final extension at 72°C for 10 min, and initial activation at 95°C for 15 min, followed by 30 cycles of denaturation at 95°C for 50 s, annealing at 50°C for 40 s, elongation at 72°C for 1 min, followed by a final extension at 72°C for 10 min. HotStarTaq Master Mix Kit (Qiagen, Hilden, Germany) and 1 μM of each primer were used for all PCR amplifications.

The PCR products were purified using either QIAquick PCR Purification Kit (Qiagen, Hilden, Germany) or ExoSAP- IT (GE Healthcare). Both strands were sequenced using the same primers as for PCRs, and sometimes internal sequencing primers were added as described by Arpin et al., Bermudes et al. and Rasheed et al. [15–17]. The BigDye® Terminator v3.1 Cycle Sequencing Kit (Applied Biosystems, Foster City, CA) were used for sequencing, followed by analysis by capillary electrophoresis with an ABI Prism 3700 DNA Analyzer (Applied Biosystems). Point mutations were accepted if present in both the forward and reverse sequences.

### Data analysis

Data were analysed using SPSS software version 20.0 (IBM SPSS statistics 20.0, SPSS Inc., Chicago, IL, USA). Chi-square test was used to determine associations between categorical variables, *p <* 0.05 was considered statistically significant.

## Results

### Prevalence of ESBL isolates

During the study period a total of 172 bacterial isolates from hospital- and community-acquired urinary tract infections were consecutively collected. The most frequent bacteria isolated were *Escherichia coli* (64%), followed by *Klebsiella pneumoniae* (15.7%) and other *Enterobacteriaceae* accounted for 20.3%. Of the 172 bacterial isolates, 23.8% (41/172) were ESBL-producers (Table 1). ESBL-producing isolates were more frequent from the hospital setting 32% (27/84) than from the community setting (16%, (14/88); *p <* 0.05). The proportion of ESBL positive *E. coli* isolates was significantly higher in hospital-acquired infections (20.3%, 11/54) compared to community-acquired infections (7.1% (4/56); *p <* 0.05). In *K. pneumoniae* isolates, ESBL-production was equally frequent in hospital- and community-acquired infections (33.3% each). The proportion of *Enterobacter cloacae* isolates producing ESBL was significantly higher in the hospital setting (71% (5/7)) than in the community setting (25% (1/4); *p >* 0.05).

**Table 1:** Distribution of ESBL positive and ESBL negative bacteria isolated from hospital- and community-acquired urinary tract infections

Bacteria spp.	Hospitalized patients			Community patients			Total
	ESBL (+)	ESBL (−)	Subtotal	ESBL (+)	ESBL (−)	Subtotal	
*E. coli*	11	43	54(64.3)	4	52	56(64)	110(64)
*K. pneumoniae*	4	8	12(14.3)	5	10	15(17)	27(15.7)
*E. cloacae*	5	2	7(8.3)	1	3	4(4.5)	11(6.4)
*C. freundii*	3	2	5(6)	1	3	4(4.5)	9(5.2)
*M. morganii*	1	1	2(2.3)	1	4	5(5.6)	7(4.1)
*P. mirabilis*	3	1	4(4.8)	2	1	3(3.3)	7(4.1)
*P. rettgeri*	0	0	0	0	1	1(1.1)	1(0.6)
Total	27	57	84(100)	14	74	88	172(100)

*HA* hospital acquired, *CA* community-acquired

### Antimicrobial susceptibility pattern

Overall, ESBL-producing isolates showed significantly higher rates of resistance towards ciprofloxacin (85.5%), doxycycline (90.2%), gentamicin (80.5%), nalidixic acid (84.5%) and trimethoprim-sulfamethoxazole (85.4%) (*p <* 0.05) compared to non-ESBL producers. All ESBL and non-ESBL-producers were susceptible to imipenem (Table 2). Multi-drug resistant was high (95%) of ESBL-producers compared to 69% of non-ESBL-producing bacteria (*p <* 0.05).

**Table 2:** Antimicrobial resistance pattern for ESBL and Non- ESBL bacteria isolates, (% of resistance isolates within each group)

Antibiotic	*E. coli*		*K. pneumoniae*		*E. cloacae*		*C. freundii*		*P. mirabilis*		*M. morganii*		*P. rettgeri*
	ESBL(+) (*n =* 15)	ESBL (−) (*n =* 95)	ESBL (+) (*n =* 9)	ESBL (−) (*n =* 18)	ESBL (+) (*n =* 6)	ESBL (−) (*n =* 5)	ESBL (+) (*n =* 4)	ESBL (−) (*n =* 5)	ESBL (+) (*n =* 5)	ESBL (−) (*n =* 2)	ESBL (+) (*n =* 2)	ESBL (−) (*n =* 5)	ESBL (−) (*n =* 1)
AMC	NA	14.7	NA	44.4	NA	80	NA	50	NA	0	NA	20	100
CTX	NA	0.0	NA	0	NA	0	NA	0	NA	0	NA	0	0
CTZ	NA	0.0	NA	0	NA	0	NA	0	NA	0	NA	0	0
CRO	NA	1.1	NA	0	NA	0	NA	0	NA	0	NA	0	0
IMP	0	0	0	0	0	0	0	0	0	0	0	0	0
CHL	26.7	37.9	88.9	50	83.3	20	75	0	100	0	100	60	100
CIP	86.7	27.4	66.7	22.2	83.3	0	50	0	100	0	100	60	0
DO	100	73.7	66.7	44.4	83.3	60	100	40	100	100	100	80	100
CN	80	18	88.9	22.2	50	0	75	0	100	0	100	20	0
NAL	86.7	37.9	66.7	27.8	75	20	100	0	100	0	100	80	100
NIT	33.3	10.5	77.8	33.3	83.3	60	75	20	100	100	100	100	100
SXT	93.3	72.6	66.7	72.2	83.3	20	75	20	100	0	100	80	0

*AMC* Amoxicillin-clavulanic acid, *CTX* cefotaxime, *CTZ* ceftazidime, *CRO* ceftriaxone, *IMP* imipenem, *CHL* chloramphenicol, *CIP* ciprofloxacin, *DO* doxycycline, *CN* gentamicin, *NAL* nalidixic acid, *NIT* nitrofurantoin, *SXT* trimethoprim-sulfamethoxazole

*NA* Not applicable

When comparing rates of resistance between HA and CA ESBL, we found that hospital-acquired *E. coli* and *K. pneumoniae* were more frequently resistant to ciprofloxacin, gentamicin and nalidixic acid than those isolated from community-acquired infections (Table 3).

**Table 3:** Antimicrobial resistance pattern of *E. coli* and *K. pneumoniae* isolates from Hospital-acquired and Community-acquired urinary tract infections (% of resistance isolates within each group)

Antibiotic	*E. coli* (*n =* 110)				*K. pneumoniae* (*n =* 27)			
	Hospital acquired		Community acquired		Hospital acquired		Community acquired	
	ESBL (+) (*n =* 11)	ESBL (−) (*n =* 43)	ESBL (+) (*n =* 4)	ESBL (−) (*n =* 52)	ESBL (+) (*n =* 4)	ESBL (−) (*n =* 8)	ESBL (+) (*n =* 5)	ESBL (−) (*n =* 10)
AMC	NA	11.6	NA	17.3	NA	62.5	NA	30.0
CTX	NA	0	NA	0	NA	0	NA	0
CTZ	NA	0	NA	0	NA	0	NA	0
CRO	NA	0	NA	0	NA	0	NA	0
IMP	0	0	0	0	0	0	0	0
CHL	27.3	41.9	25.0	34.6	75.0	62.5	100	40.0
CIP	90.9	32.6	75.0	23.1	100	25.0	40.0	20.0
DOX	100	69.8	100	76.9	75.0	62.5	60.0	30.0
CN	90.9	16.3	50.0	21.2	100	37.5	80.0	10.0
NAL	90.0	41.9	75.0	34.6	100	25.0	40.0	30.0
NIT	27.3	27.3	50.0	50.0	100	100	60.0	60.0
SXT	90.9	62.8	100	80.8	75.0	87.5	60.0	60.0

(+) = positive; (−) = negative

*AMC* Amoxicillin-clavulanic acid, *CTX* cefotaxime, *CTZ* ceftazidime, *CRO* ceftriaxone, *IMP* imipenem, *CHL* chloramphenicol, *CIP* ciprofloxacin, *DO* doxycycline, *CN* gentamicin, *NAL* nalidixic acid, *NIT* nitrofurantoin, *SXT* trimethoprim-sulfamethoxazole

Non-ESBL-producing *E. coli* and *K. pneumoniae* from hospital- and community-acquired infections were less frequently resistant to gentamicin, nalidixic acid and ciprofloxacin. However, non-ESBL-producing *E. coli* from outpatients showed moderately high rates of resistance to trimethoprim-sulfamethoxazole, nitrofurantoin and doxycycline compared to isolates from inpatients. Higher rates of resistance to amoxicillin-clavulanic acid and trimethoprim-sulfonamide were observed in hospital-acquired as compared to community-acquired isolates of non-ESBL-producing *K. pneumoniae*. (Susceptibility profile for other isolates see Additional file 1: Table S1).

### Molecular characterization of ESBL producing bacteria

An ESBL genotype could be identified for 32 of the all ESBL confirmed isolates (Table 4). Among these, 90.6% (29/32) were *bla*_CTX-M-15_ positive, 6.25% (2/32) were *bla*_SHV-12_ positive and one isolate was found to carry both *bla*_CTX-M-15_ and *bla*_SHV-12_. None of the isolate carried *bla*_TEM_. Of the 29 isolates carrying *bla*_CTX-M-15_, 69% (20/29) were hospital isolates and 31% (9/29) from community settings. (All three isolates harboring *bla*_SHV-12_ were from hospital setting (*E. coli*, *E. cloacae* and *Citrobacter freundii*). All isolates carrying *bla*_CTX-M-15_ displayed high rates of resistance to non β-lactam agents, including ciprofloxacin (88%), gentamicin (81.5%) and trimethoprim-sulfamethoxazole (89%).

**Table 4:** ESBL genotypes in bacteria isolated from hospital-acquired and community-acquired urinary tract infections

Bacteria spp.	CTX-M-15			SHV-12			CTX-M-15/SHV-12	
	HA	CA	Subtotal	HA	CA	Subtotal	HA	CA
*E. coli*	10	4	14	0	0	0	1	0
*K. pneumoniae*	4	3	7	0	0	0	0	0
*E. cloacae*	3	1	4	1	0	1	0	0
*C. freundii*	2	0	2	1	0	1	0	0
*M. morganii*	1	1	2	0	0	0	0	0
Total	20	9	29	2	0	0	1	0

*HA* Hospital acquired, *CA* community acquired

## Discussion

In recent years there has been an alarming increase in community acquired infections with ESBL-producing bacteria [5, 12, 18]. Spread of these strains in the community is a major concern to patient healthcare, since most display multidrug resistance, limiting outpatient treatment options. The resultant increasing use of broad-spectrum antibiotics to treat infections caused by ESBL-producers is expected to lead to further emergence of antimicrobial resistance. However, little data exist on molecular characterization of ESBL isolates causing community-acquired urinary tract infections in Tanzania and Africa. The current study shows that ESBL-producing isolates caused both community and hospital-acquired urinary tract infections in 2004, when CTX-M-15 was first reported in Tanzania.

The overall frequency of ESBL-producing *Enterobacteriaceae* among urinary tract pathogens in this study was 23.8%. The frequency of ESBL-producing pathogens was significantly higher in hospital-acquired compared to community-acquired uropathogens. Our finding is in agreement with other studies [19–22] reporting higher frequency of ESBL-producers in hospital-acquired urinary tract infections compared to community-acquired infections. A possible explanation for this could be that hospital-acquired infections were more likely associated with prolonged hospitalization, comorbidities, previous antibiotic use and urinary catheterization which are well-known risk factors for acquisition of ESBL-producing pathogens [19]. However, the finding of ESBL-producing isolates in community urinary infections is worrisome because of the limited treatment options, considering most of these isolates display multidrug resistance.

Similar to other studies in Africa [4, 10, 22], we found that ESBL-producing isolates from both hospital and community settings displayed high rates of resistance to ciprofloxacin, trimethoprim-sulfamethoxazole, gentamicin, nalidixic acid and doxycycline. Resistance to commonly prescribed oral antimicrobials in these resource-limited settings, specifically to ciprofloxacin and trimethoprim-sulfamethoxazole, limits outpatient therapeutic options. Considering that most of the outpatients present with uncomplicated urinary tract infections, opting to injectable and expensive antimicrobials increases health-care burdens. We also found non-ESBL-producing *E. coli* from community-acquired urinary tract infections had moderate to high rates of resistance to trimethoprim-sulfamethoxazole, doxycycline and nitrofurantoin. This could be expected, since oral antimicrobials are inexpensive and easily available over the counter, and self-treatment is common in Africa; these are well known factors driving emergence of antimicrobial resistance bacteria.

Among 41 ESBL defined isolates, 78% were found to carry ESBL genes. Our finding of a predominance of CTX-M-15 is in line to previous and recent studies from hospital and community urinary tract infections [6, 7, 18, 23], and our finding concurs with those of studies from the same setting, which found CTX-M-15 as the dominant ESBL genotype [8–10, 24]. CTX-M types ESBLs, in particular CTX-M-15, are known for their rapid dissemination world-wide among the members of *Enterobacteriaceae* [6, 12, 23, 25, 26]. It has also been suggested that the widespread use of ceftriaxone and cefotaxime could be a reason of emergence and spread of CTX-M enzymes [27].

Our study had some limitations; one our isolates were collected in 2004 and may not imply the current situation. However, our findings shed lights on community spread of ESBL-producers and suggest existence in Tanzania at least since 2004. Second being a laboratory-based study, clinical information was not obtained, and we could not analyze risk factors for ESBL infections. Furthermore, epidemiological typing to assess clonality of the isolates was not performed, and this could have added value to the understanding of the epidemiological spread of ESBL genes.

## Conclusion

In conclusion, we report the presence of *Enterobacteriaceae* harboring CTX-M-15 type ESBL causing community-acquired urinary tract infections in Tanzania as early as 2004. Furthermore, both ESBL and non-ESBL-producing isolates displayed high rates of multidrug resistance. Further investigation needs to be performed to understand the transmission dynamics of CTX-M type of ESBL resistance.

## Additional file

Additional file 1: Table S1. Antimicrobial resistance pattern of isolates from Hospital-acquired and Community-acquired urinary tract infections (% of resistance isolates within each group). (DOC 52 kb)
