# Supplementary material for: Detection of CTX-M-15 beta-lactamases in Enterobacteriaceae causing hospital- and community-acquired urinary tract infections as early as 2004, in Dar es Salaam, Tanzania
Source: BMC Infect Dis. 2017 Apr 17;17:282. doi: 10.1186/s12879-017-2395-8 (PMC5392921; doi:10.1186/s12879-017-2395-8)
Supplement: Additional file 1: Table S1. — Antimicrobial resistance pattern of isolates from Hospital-acquired and Community-acquired urinary tract infections (% of resistance isolates within each group). (DOC 52 kb) [file 12879_2017_2395_MOESM1_ESM.doc]

**Supplementary table 1: Antimicrobial resistance pattern of isolates from Hospital-acquired and Community-acquired urinary tract infections (% of resistance isolates within each group)**

| Antibiotics | *E. cloacae* (11) | | | | *C. freundii* 9 | | | | *P. mirabilis* (7) | | | | *M. morganii* (7) | | | |
| --- | --- | --- | --- | --- | --- | --- | --- | --- | --- | --- | --- | --- | --- | --- | --- | --- |
|  | HA | | CA | | HA | | CA | | HA | | CA | | HA | | CA | |
|  | ESBL+ | ESBL- | ESBL + | ESBL- | ESBL + | ESBL- | ESBL + | ESBL - | ESBL+ | ESBL - | ESBL + | ESBL - | ESBL+ | ESBL - | ESBL+ | ESBL - |
|  |  |  |  |  |  |  |  |  |  |  |  |  |  |  |  |
| AMC | NA | 20 | NA | 50 | NA | 50 | NA | 100 | NA | 0 | NA | 0 | NA | 0 | NA | 0 |
| CTX | NA | 0 | NA | 0 | NA | 0 | NA | 0 | NA | 0 | NA | 0 | NA | 0 | NA | 0 |
| CTZ | NA | 0 | NA | 0 | NA | 0 | NA | 0 | NA | 0 | NA | 0 | NA | 0 | NA | 0 |
| CRO | NA | 0 | NA | 0 | NA | 0 | NA | 0 | NA | 0 | NA | 0 | NA | 0 | NA | 0 |
| IMP | 0 | 0 | 0 | 0 | 0 | 0 | 0 | 0 | 0 | 0 | 0 | 0 | 0 | 0 | 0 | 0 |
| CHL | 80 | 100 | 100 | 33.3 | 100 | 50 | 100 | 33.3 | 100 | 100 | 100 | 0 | 100 | 100 | 100 | 100 |
| CIP | 80 | 50 | 100 | 0 | 66.7 | 0 | 100 | 0 | 100 | 0 | 100 | 0 | 100 | 75 | 75 | 0 |
| DOX | 80 | 50 | 100 | 0 | 66.7 | 0 | 100 | 33.3 | 100 | 0 | 100 | 0 | 100 | 100 | 100 | 100 |
| CN | 80 | 0 | 100 | 0 | 33.3 | 0 | 100 | 0 | 100 | 75 | 100 | 0 | 100 | 0 | 100 | 0 |
| NAL | 40 | 0 | 100 | 0 | 66.7 | 40 | 100 | 100 | 100 | 0 | 100 | 0 | 100 | 25 | 100 | 0 |
| NIT | 100 | 0 | 100 | 0 | 66.7 | 50 | 100 | 0 | 100 | 0 | 100 | 0 | 100 | 100 | 100 | 0 |
| SXT | 100 | 100 | 100 | 100 | 66.7 | 66.7 | 100 | 100 | 100 | 100 | 100 | 100 | 100 | 100 | 100 | 100 |
|  |  |  |  |  |  |  |  |  |  |  |  |  |  |  |  |  |

Note: P. rettgeri was 1 isolate and 100% resistant to AMC, CHL and NAL. (+) = positive; (-) = negative; AMC=Amoxicilln-clavulanate; CTX= cefotaxime; CTZ=ceftazidime CRO = ceftriaxone; IMP=imipenem; CHL=chloramphenicol; CIP=ciprofloxacin; DOX=doxycycline GEN=gentamicin; NAL=nalidixic acid; NIT=nitrofurantoin; SXT=trimethoprim-sulfamethoxazole
